# PTPRO knockdown protects against inflammation in hemorrhage shock-induced lung injury involving the NF-κB signaling pathway

**DOI:** 10.1186/s12931-022-02118-2

**Published:** 2022-07-29

**Authors:** Zhirong Huan, Ying Tang, Ce Xu, Jimin Cai, Hao Yao, Yan Wang, Fanyu Bu, Xin Ge

**Affiliations:** 1 Department of ICU, Wuxi 9th Affiliated Hospital of Soochow University, Wuxi, Jiangsu 214000 People’s Republic of China; 2 Department of Biology, School of Life Sciences, Southern University of Science and Technology, Shenzhen, 518055 Guangdong China; 3 Department of Reconstruction Surgery, Wuxi 9th Affiliated Hospital of Soochow University, Wuxi, Jiangsu 214000 People’s Republic of China; 4 Orthopedic Institution of Wuxi City, Wuxi, 214000 Jiangsu China; 5 Department of ICU, The First Affiliated Hospital of Harbin Medical University, Harbin, 150001 Heilongjiang People’s Republic of China

**Keywords:** Protein tyrosine phosphatase receptor type O (PTPRO), Hemorrhage shock (HS), Lung injury, Inflammation, The NF-κB signaling pathway

## Abstract

**Background:**

Hemorrhage shock (HS) is characterized by decreased tissue oxygenation and organ damage due to severe blood loss. Protein tyrosine phosphatase receptor type O (PTPRO) is abnormally up-regulated in the rat lungs after trauma/HS.

**Methods:**

To elucidate the regulatory mechanism of PTPRO in lung inflammation following HS, we established a rat model of HS via withdrawing blood by a catheter inserted into the femoral artery followed by resuscitation. The rats were infected with lentivirus harboring short hairpin RNA (shRNA) targeting PTPRO by intratracheal instillation.

**Results:**

PTPRO was significantly up-regulated in rat lungs after HS. PTPRO knockdown enhanced epithelial integrity and reduced capillary leakage by up-regulating tight junction proteins zonula occludens-1 (ZO-1) and occludin (OCC) in the lungs. Besides, HS-induced myeloperoxidase activity and inflammatory cell infiltration was mitigated by PTPRO knockdown. The expression of inflammatory cytokines/chemokines (TNF-α, IL-6, MIP-2, MCP-1, and KC) in the lungs and bronchoalveolar lavage fluid was regressed after PTPRO knockdown. The nuclear factor kappa B (NF-κB) pathway was involved in HS-induced lung inflammation. PTPRO down-regulation inhibited the NF-κB pathway activation by suppressing the phosphorylation of NF-κB and its translocation from the cytoplasm into the nucleus in HS.

**Conclusion:**

Taken together, we demonstrated that PTPRO knockdown may contribute to attenuating inflammation in HS-induced lung injury via inhibiting NF-κB pathway activation.

**Supplementary Information:**

The online version contains supplementary material available at 10.1186/s12931-022-02118-2.

## Introduction

Hemorrhagic shock (HS), a critical disease with a high mortality rate, occurs in patients with uncontrolled bleeding including trauma, maternal hemorrhage, and gastrointestinal hemorrhage, resulting in a circulatory dysfunction leading to decreased tissue oxygenation, organ damage, and even death [1–4]. About 1.9 million deaths result from HS per year worldwide [5]. HS initiates the inflammatory responses which facilitate organ injury and early multiple organ failure [6]. Acute respiratory distress syndrome (ARDS) is an important organ dysfunction syndrome caused by trauma/HS [7]. HS contributes to the development of ALI through exacerbating inflammation and subsequent immunosuppression in the lungs, which results in nosocomial infections and secondary complications, thus leading to the final lung dysfunction and mortality [4].

Protein tyrosine phosphatase receptor type O (PTPRO) is an integral membrane protein belonging to the phosphor tyrosine phosphatases (PTPs) family and regulating tyrosine phosphorylation in cells [8]. In adult tissues, PTPRO is widely expressed in the lung, heart, brain, and other organs [9]. According to the previous study, PTPRO aggravates atherosclerosis by promoting oxidative stress and cell apoptosis induced by oxidized low-density lipoprotein [10]. Oestrogen restricts renal podocyte apoptosis by inhibiting PTPRO [11]. Besides, PTPRO is a candidate tumor suppressor in human lung cancer and down-regulation of PTPRO mediated by miR-6803-5p promotes the proliferation and invasion of the cancer cells in colorectal cancer [12, 13]. Recently, more and more researchers focus on the role of PTPRO in inflammation. For instance, PTPRO has an inflammation promotion effect on fulminant hepatitis and ulcerative colitis [14, 15]. PTPRO is up-regulated in placental mononuclear cells in patients with preeclampsia and its down-regulation mediated by miR-548c-5p overexpression plays an anti-inflammatory role in preeclampsia [16]. In the lungs, the role of PTPRO has only been reported in human lung cancer. According to the information retrieved from the Gene Expression Omnibus (GEO) DataSets (https://www.ncbi.nlm.nih.gov/gds), PTPRO is abnormally up-regulated in the rat lungs at 3 h post-trauma/HS (GSE6332). However, the functions of PTPRO in lung inflammation following HS are poorly elucidated.

The nuclear factor kappa B (NF-κB) pathway is considered a pro-inflammatory signaling pathway [17]. This pathway is strongly involved in the systemic inflammatory changes and immune response following hemorrhage shock [18]. Ethyl pyruvate restrains systemic leukocyte activation via regulating NF-κB after HS [19]. Inhibition of the activation of NF-κB reduces circulatory failure and organ injury and dysfunction in HS [20]. According to these findings, the reduction of the NF-κB pathway activation contributes to attenuating HS-induced inflammation and organ injury.

A series of studies have reported that PTPRO aggravates inflammation by activating the NF-κB signaling pathway [10, 14, 15]. There is little information available in the literature about the role of PTPRO in HS-induced inflammation and lung injury. Based on the previous results, we hypothesized that PTPRO functions in ALI following HS via regulating the NF-κB signaling pathway. We conducted this study to explore the expression and potential regulatory mechanism of PTPRO in lung inflammation following HS, which may contribute to the understanding of ALI pathogenesis following HS and indicate PTPRO as a potential therapeutic target for attenuating lung dysfunction induced HS.

## Materials and methods

### Animal models

Twelve-week-old Sprague–Dawley rats purchased from Liaoning Changsheng Biotech Biotech Co., Ltd. (Benxi, China) were used for the HS model. The rats were allowed free access to food and water under a condition of 22 ± 1 °C and 12-h light/dark cycle for a 1-week acclimation period. A fixed-pressure hemorrhagic shock and resuscitation protocol was used to establish a rat model of HS as previously reported [21–23]. Briefly, the femoral artery was cannulated with a catheter and a blood pressure transducer was connected for continuous blood pressure monitoring during the modeling. Blood was withdrawn from the arterial catheter until mean arterial pressure (MAP) reached 40–50 mmHg and allowed to maintain at this level for 1 h. Additional bloodletting might be necessary to maintain MAP during this hemorrhage period. After 1-h hemorrhage, the rats were resuscitated with warm Lactated Ringer’s solution via intravenous delivery for blood pressure restoration (to 100 mmHg, systolic blood pressure) and the blood pressure was maintained for 3 h. The fluid administered during resuscitation was about four times the volume of the total blood removed (9 mL), so its volume was about 36 mL for each rat and the rate of fluid infusion was 0.6 mL/min. The rats that underwent HS surgery were the similar age and size, and the volume of resuscitation solution did not differ between groups. Three hours after resuscitation, all rats were euthanized for tissue collection. The sham-operated rats underwent the same catheter implantation procedure but without bloodletting or resuscitation. The body temperature of the rats was maintained at 37 °C during the surgery. For knocking down PTPRO in the rats, 48 h before hemorrhage by a catheter inserted into the femoral artery, the rat was infected with 1 × 10^8^ TU lentivirus encoding short hairpin RNA (shRNA) sequence targeting PTPRO (LV-shPTPRO) or its negative control shRNA (LV-shNC) by intratracheal instillation through the mouth. The rats were categorized into four groups (n = 30 per group): the Sham group, the HS group, the HS + LV-shNC group, and the HS + LV-shPTPRO group. For each group, 12 rats were used for Evans blue dye extravasation, 6 for histological analysis and ELISA assays, and 6 for PCR and western blotting, and 6 for BALF cell counts. This study was performed following the Institution Animal Care and Use Committee of Wuxi 9th Affiliated Hospital of Soochow University. After euthanasia, the blood, bronchoalveolar lavage fluid (BALF), and lung tissues were collected and stored at − 70 °C or fixed with 4% paraformaldehyde.

### Clinical study

A clinical study was conducted in 7 patients with severe HS after polytrauma from Wuxi 9th Affiliated Hospital of Soochow University. Thirty healthy participants served as the controls. The blood samples were collected from all subjects after obtaining written informed consent. At the time of blood sampling, those HS patients were under HS in ICU. Details of patient characteristics were included in Additional file 1: Table S1. The study was performed in accordance with the Declaration of Helsinki and approved by the Ethics Committee of the Wuxi 9th Affiliated Hospital of Soochow University (KT2020029).

### Capillary leakage

Evans blue (EB) dye extravasation was performed to assess capillary leakage in the lungs and BALF. Twenty minutes before euthanasia, the rats were subjected to the tail vein injection with EB dye (50 mL/kg; Aladdin, Shanghai, China). After being euthanized, the heart was perfused with saline to remove the redundant dye in the vessels. EB was extracted from the lung tissues by incubation in formamide (4 m/g tissue; Aladdin, Shanghai, China) at 60 °C for 48 h. The supernatant was detected with a microplate reader (800TS; BioTek, Winooski, VT, USA) at 620 nm after centrifugation. EB content was calculated according to a standard curve of EB in formamide and used to represent extravasation.

### Enzyme-linked immunosorbent assay (ELISA)

The levels of tumor necrosis factor α (TNF-α), interleukin (IL)-6, macrophage inflammatory protein (MIP)-2, monocyte chemoattractant protein (MCP)-1, and keratinocyte chemoattractant (KC) in BALF were detected with respective specific ELISA kits (LIANKE Biotech, Hangzhou, China) according to the manufacturer's instructions.

### Quantitative real-time PCR

Using the Total RNA Isolation Kit (Tiangen Biotech Co. Ltd., Beijing, China) and BeyoRT II M-MLV reverse transcriptase (Beyotime, Shanghai, China), total RNA was isolated from the lungs and reverse transcribed into the first cDNA. A quantitative PCR assay was carried out by Exicycler™ 96 Real-time PCR System (Bioneer Corporation, Daejeon, Korea) using SYBR Green (Solarbio, Beijing, China). The level of mRNA was normalized to GAPDH. The primers were synthesized by Genscript Biotechnology Co., Ltd. (Nanjing, China), and the sequences were shown as follows: human PTPRO forward 5′-CTGACCTGCCAGAAACAA-3′, reverse 5′-AGGACCCAAAGGATAGAG-3′; rat PTPRO forward 5′-TGCTCGGGCTCTTTGTGC-3′, reverse 5′-ATCGGGATGGTTTGGTGA-3′; TNF-α forward 5′-CGGAAAGCATGATCCGAGAT-3′, reverse 5′-AGACAGAAGAGCGTGGTGGC-3′; IL-6 forward 5′-AACTCCATCTGCCCTTCA-3′, reverse 5′-CTGTTGTGGGTGGTATCCTC-3′; KC forward 5′-ACCCAAACCGAAGTCATAGC-3′, reverse 5’-GGGACACCCTTTAGCATCTT-3′; MCP-1 forward 5′-CTGTCACGCTTCTGGG-3′, reverse 5′-GCCGACTCATTGGGAT-3′; MIP-2 forward 5′-ACTGGTCCTGCTCCTCCT-3′, reverse 5′-TTAGCCTTGCCTTTGTTC-3′; OCC forward 5′-CAGAGCCTATGGAACGG-3′, reverse 5′-CAAGGAAGCGATGAAGC-3′; ZO-1 forward 5′-ATCTCCAGTCCCTTACCTTTC-3′, reverse 5′-TGGTGCTCCTAAACAATCAG-3′.

### Western blot analysis

The lung tissues separated from the rats were lysed using RIPA buffer (Solarbio, Beijing, China) supplemented with 1 mM phenylmethylsulfonyl fluoride (PMSF; Solarbio, Beijing, China) to isolate total proteins. Nuclear proteins were extracted using Nuclear Protein Extraction Kit (Solarbio, Beijing, China) according to the manufacturer's instructions. Protein quantified by bicinchoninic acid (BCA) Kit (Solarbio, Beijing, China) was separated by sodium dodecyl sulfate–polyacrylamide gel electrophoresis (SDS–PAGE). After electrophoresis, proteins were transferred onto a polyvinylidene difluoride membrane (PVDF; Millipore, Billerica, MA, USA). The membrane was blocked with 5% skim milk (Sangon Biotech, Shanghai, China) for 1 h. Afterward, the membrane was incubated with the primary antibody including PTPRO antibody (1:500 dilution; Affinity, Cincinnati, OH, USA), ZO-1 antibody (1:500 dilution; Affinity, Cincinnati, OH, USA), OCC (1:500 dilution; Affinity, Cincinnati, OH, USA), p-IKKα/β antibody (1:2000 dilution; Affinity, Cincinnati, OH, USA), IKKα/β antibody (1:1000 dilution; Affinity, Cincinnati, OH, USA), p-P65 (S536) antibody (1:1000 dilution; ABclonal, Shanghai, China), NF-κB (P65) antibody (1:500 dilution; ABclonal, Shanghai, China), GAPDH antibody (1:10,000 dilution; Proteintech, Rosemont, IL, USA), and Histone H3 antibody (1:5000 dilution; GeneTex, Alton PkwyIrvine, CA, USA) overnight at 4 °C, followed by incubation with horseradish peroxidase-linked goat anti-rabbit or anti-mouse secondary antibodies (1:3000 dilution; Solarbio, Beijing, China) at 37 °C for 1 h. Protein bands were visualized using electrochemiluminescence (ECL) reagent (Solarbio, Beijing, China). Band intensities were analyzed with Gel-Pro-Analyzer software and normalized to band intensity of GAPDH or Histone H3.

### Histologic analysis

Histological changes in the lungs were detected with hematoxylin and eosin (H&E) staining. The lung tissues fixed with 4% paraformaldehyde were embedded in paraffin and cut into 5-μm sections. After dewaxing and rehydration, the slides were stained with hematoxylin (Solarbio, Beijing, China) and eosin (Sangon Biotech, Shanghai, China) or PTPRO antibody (1:50 dilution; Santa Cruz, Shanghai, China) with DAPI counterstaining. The images were captured under a microscope (BX53, Olympus, Tokyo, Japan) at 200 × magnification. The assessment of lung injury was based on 3 parameters (alveolar thickness, capillary red cell retention, and leukocyte infiltration) using a 3-point score system [24], respectively: 0 indicates no pathologic alteration, 1 indicates mild, 2 indicates moderate, 3 indicates severe. The pathologic value for each field was the sum of these 3 parameters’ score. Six random fields were assessed for each group and its values averaged.

### Immunofluorescence staining

The lung tissue slides embedded in paraffin were exposed to citrate solution for antigen retrieval after dewaxing and rehydration. The slides were blocked with goat serum (Solarbio, Beijing, China) at room temperature for 15 min and incubated with MPO antibody (1:100 dilution; ABclonal, Shanghai, China) or P65 antibody (1:100 dilution; ABclonal, Shanghai, China) overnight at 4 °C. For double immunofluorescence staining, the slides were probed with PTPRO (1:50 dilution, Santa, USA) and keratin 19 (1:100 dilution; ABclonal, Shanghai, China) or beta IV Tublin (1:100 dilution; Affinity, Cincinnati, OH, USA). Then the tissues were incubated in the dark with a Cy3-conjugated goat anti-rabbit IgG (1:200 dilution; Beyotime, Shanghai, China) or FITC-labeled goat anti-mouse IgG (1:200 dilution; Abcam, Cambridge, UK) at room temperature for 1 h. The tissues were re-stained with 4′,6-diamidino-2-phenylindole (DAPI; Beyotime, Shanghai, China), and the images were captured at 400 × magnification by a fluorescence microscope (BX53, Olympus, Tokyo, Japan).

### Myeloperoxidase (MPO) activity

Infiltration of neutrophils into the lung tissue was assessed by MPO activity. Myeloperoxidase assay kit (Jiancheng Bioengineering Institute, Nanjing, China) was used for the detection of MPO activity in the lungs according to the manufacturer's instructions.

### BALF cell counts

After centrifugation and re-suspension in phosphate buffered saline, total leukocyte was counted using a hemocytemeter under a microscope. To analyze differential count of cells in BALF, cell specimens were prepared on glass slides using cell smear method and stained with Giemsa dye (Jiancheng Bioengineering Institute, Nanjing, China). The number of neutrophils and macrophage was determined manually under a microscope (Olympus, Tokyo, Japan) according to the standard morphological criteria.

### Statistical analysis

The data were analyzed using GraphPad Prism 7.0 and expressed as mean ± standard deviations (SD) with 6 biological repetitions (n = 6). The values of different groups were compared with one-way analysis of variance (ANOVA) followed by Tukey’s test. Significance was considered when P < 0.05 between different groups.

## Results

### HS strongly induces the expression of PTPRO in human serum and rat lungs

**Fig. 1:**
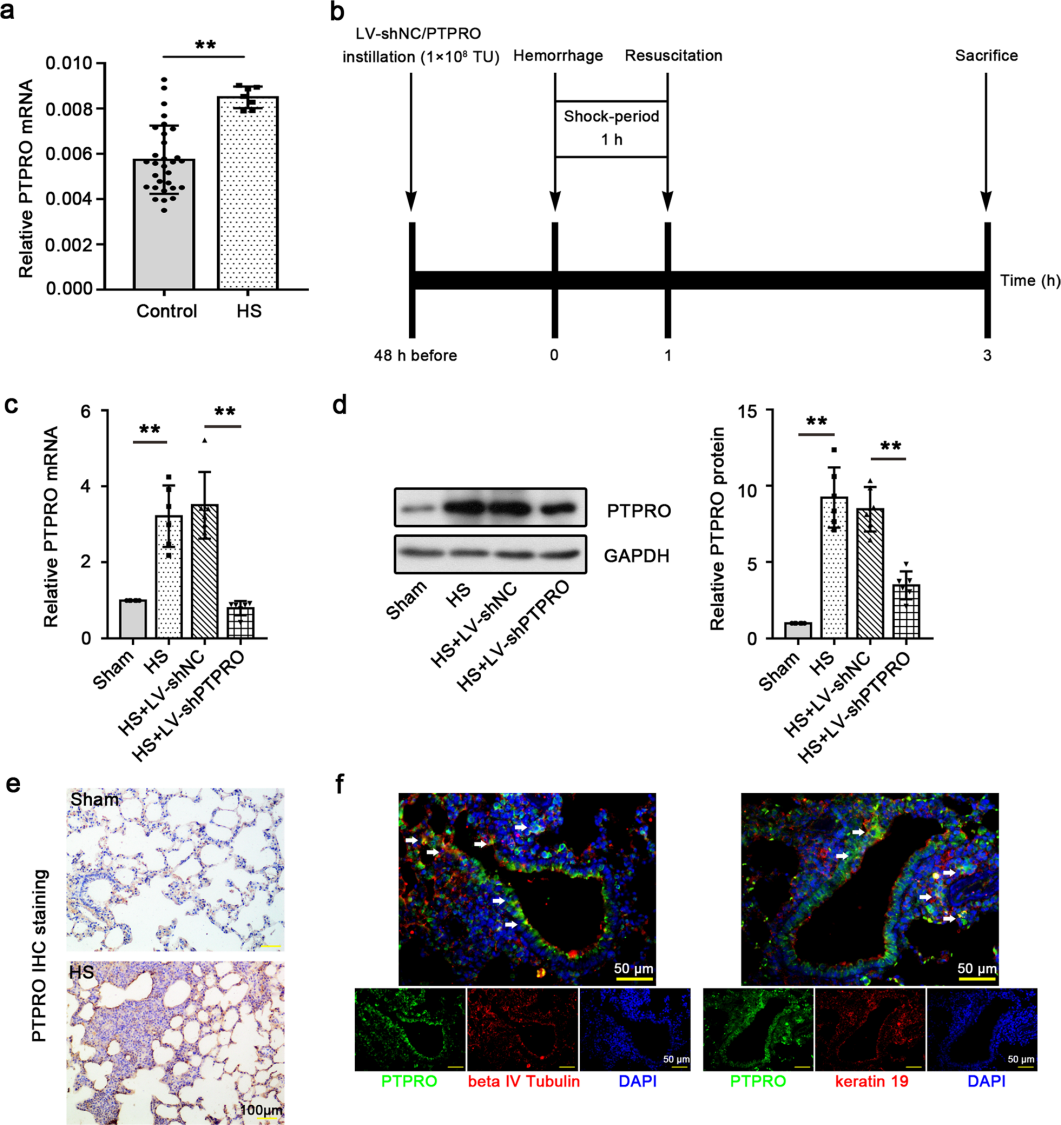
**a** Increased expression of PTPRO in the serum of HS patients and lungs of HS rats. RT-qPCR analysis of the expression of PTPRO in the serum of HS patients (n = 7) versus healthy participants (n = 30). **b** Timeline of the experiments. Rats were subjected to bloodletting by a catheter inserted into the femoral artery. Mean arterial pressure was maintained at 40–50 mmHg for 1 h (shock-period) and then the rats were resuscitated. Three hours after resuscitation, all rats were euthanized for tissue collection. For PTPRO knockdown, the rats were instilled with lentivirus harboring short hairpin RNA (shRNA) targeting PTPRO (LV-shPTPRO) or negative control shRNA (LV-shNC) 48 h before hemorrhage. **c, d** HS strongly induces the expression of PTPRO in rat lungs. **c** The relative mRNA expression of PTPRO in the lung of HS rats was detected by RT-qPCR. **d** Representative blot image and relative PTPRO protein expression in rat lungs were detected by Western blot. **e** Immunohistochemical staining for PTPRO in rat lungs. Magnification: 200 ×. Scale = 100 μm. **f** Double immunofluorescence staining of PTPRO and beta IV tubulin or keratin 19. Magnification: 400 × . Scale = 50 μm. White arrows show the representative co-localization. The values represent the mean (n = 6) ± SD. **P < 0.01

The serum samples were collected from 7 HS patients and 30 healthy participants and PCR analysis for PTPRO expression was performed. PTPRO was shown to be highly expressed in the serum of HS patients (Fig. 1a). Abnormalities in the expression suggests a potential role of PTPRO in HS pathogenesis, and thus we further investigated PTPRO expression and function in the lung of rats received experimental HS (Fig. 1b). As shown in Fig. 1c, d, HS rats exhibited higher PTPRO expression in the lungs at both mRNA and protein levels compared to sham-operated rats. After injection of lentivirus harboring PTPRO shRNA, HS rats displayed a significantly decreased lung expression of PTPRO. These indicated that HS strongly induced PTPRO expression in rat lungs and lentivirus-mediated shRNA interference successfully knocked down this gene in the rats. Histological analysis was performed to further investigate the protein expression and distribution of PTPRO in the lung of rats with experimental HS. Immunohistochemical staining showed an upregulation of PTPRO in HS rat lungs with severe pathological injury (Fig. 1e). Besides, double immunofluorecence staining revealed the co-localization of PTPRO with several major epithelial cell markers including beta IV tubulin for ciliated cells and keratin 19 for alveolar epithelial cells (Fig. 1f). The expression of PTPRO in lung epithelial cells provides insights into its function in HS.

### Knockdown of PTPRO reduces capillary leakage and attenuates HS-induced lung injury

**Fig. 2:**
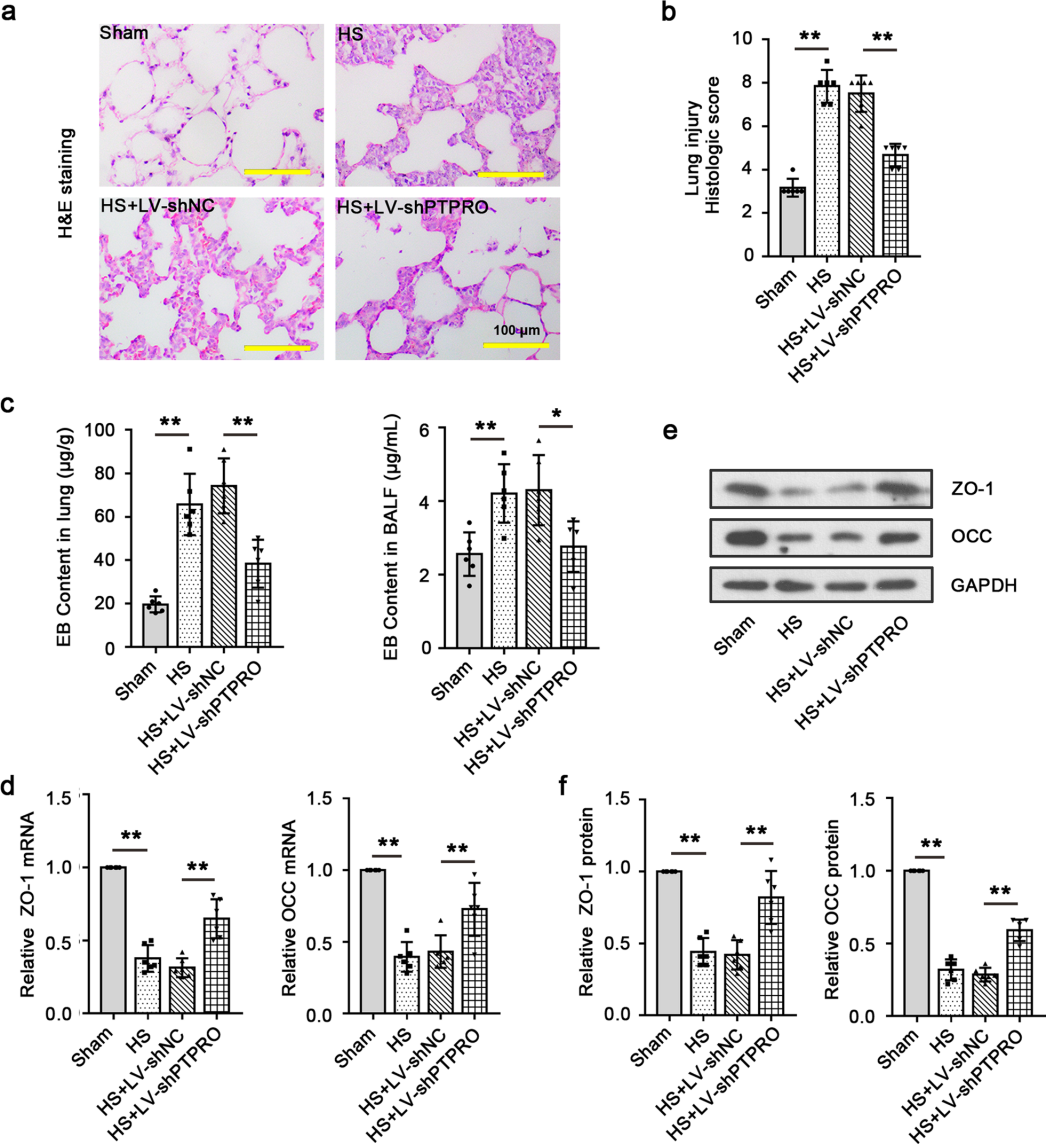
Kockdown of PTPRO reduces capillary leakage and attenuates HS-induced lung injury. **a** Lung histopathology in the HS model was assessed by H&E staining. Magnification: 200 × . Scale = 100 μm. **b** Histologic score corresponding to H&E staining for lung injury assessment. **c** Micrograms of Evans blue (EB) per gram of lung tissue and per microlitre of bronchoalveolar lavage fluid (BALF) were used to assess lung permeability in HS model. **d** Relative mRNA levels of tight junction proteins zonula occludens-1 (ZO-1) and occludin (OCC) in the lung tissues were determined by RT-qPCR. **e**, **f** Representative blot image **e.** and relative protein expression **f** of ZO-1 and OCC in the lung tissues were detected by Western blot. Values are means (n = 6) ± SD. **P < 0.01

The severity of the lung injury induced by HS was detected with H&E staining. As the results showed, alterations in the alveolar-capillary barrier, more compact lung parenchyma, alveolar septal thickening, and inflammatory cell infiltration occurred in the lungs due to HS, which were significantly attenuated by LV-shPTPRO (Fig. 2a). Histologic score that corresponds to H&E staining suggested that HS induced severe lung injury in rats and knockdown of PTPRO prevented it (Fig. 2b). The content of EB dye via intravenous injection was detected in the lung tissues and BALF for lung permeability assessment. EB content in the lungs and BALF of HS rats was dramatically increased compared to those of sham-operated rats and it was significantly reduced when PTPRO expression was repressed (Fig. 2c). Moreover, the expression of tight junction proteins zonula occludens-1 (ZO-1) and occludin (OCC), biomarkers of epithelial integrity, was markedly decreased in the lung by HS, while it was partially restored after PTPRO knockdown (Fig. 2d–f). These findings suggest that down-regulation of PTPRO enhances the epithelial integrity and reduces HS-induced capillary leakage through up-regulating ZO-1 and OCC in the lungs.

### Down-regulation of PTPRO decreases inflammatory cell infiltration and related cytokine/chemokine levels in HS

**Fig. 3:**
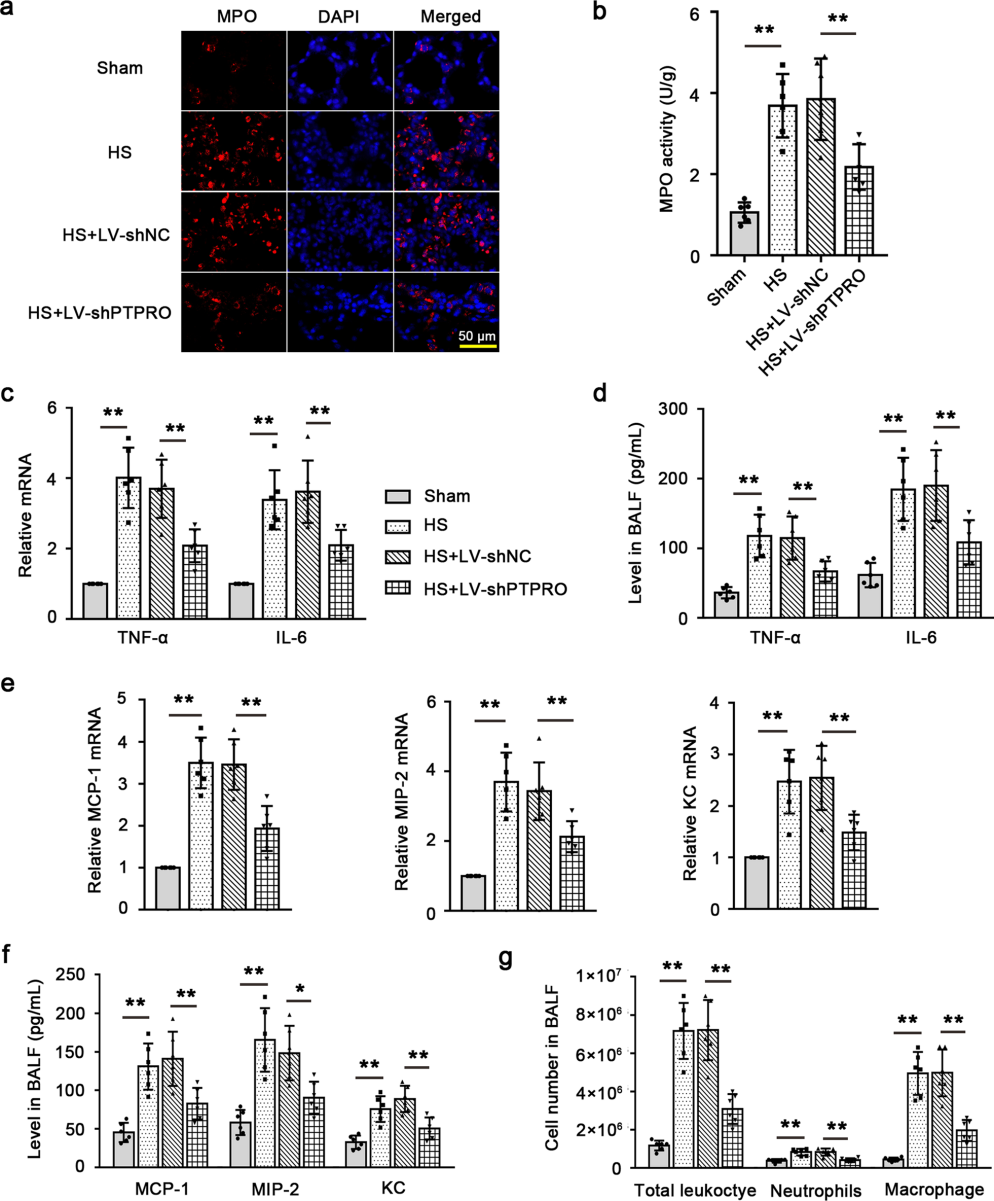
Down-regulation of PTPRO decreases inflammatory cell infiltration and related cytokine/chemokine levels in HS. **a** Representative images of myeloperoxidase (MPO) immunofluorescence staining in the lung of HS rats were shown. Magnification: 400 × . Scale = 50 μm. **b** MPO activity in the lung tissues of HS rats was detected with the MPO kit. **c** Relative mRNA levels of pro-inflammatory cytokines TNF-α and IL-6 were detected by RT-qPCR. **d** The levels of TNF-α and IL-6 in bronchoalveolar lavage fluid (BALF) were detected with ELISA. **e** Relative mRNA levels of chemokine MCP-1, MIP-2, and KC in the lung tissues. **f** The levels of MCP-1, MIP-2, and KC in BALF were detected with ELISA. **g** BALF cell counts including total leukocyte, neutrophils, and macrophage. Values are means (n = 6) ± SD. *P < 0.05, **P < 0.01

Immunofluorescence staining and activity measurement of MPO showed that the expression and activity of MPO were enhanced in the lungs by HS. PTPRO knockdown markedly reduced post-shock MPO expression and activity, indicating that HS-induced neutrophil infiltration into the lungs was mitigated by PTPRO expression suppression (Fig. 3a, b). Therefore, down-regulation of PTPRO could alleviate HS-induced neutrophil infiltration in rat lungs. The expression of inflammatory cytokine or chemokine including TNF-α, IL-6, MIP-2, MCP-1, and KC in the lung tissues was analyzed. As shown in Fig. 3c, the mRNA levels of pro-inflammatory cytokine TNF-α and IL-6 were significantly up-regulated in the lungs of HS rats compared to those of sham-operated rats, while they were regressed by suppression of PTPRO. The levels of TNF-α and IL-6 in BALF exhibited similar alterations as their pulmonary mRNA expression (Fig. 3d). Furthermore, HS increased the expression of inflammatory chemokines including MCP-1 (a chemoattractant to macrophages) and KC and MIP-2 (chemoattractants to neutrophils) in the lungs (Fig. 3e) as well as their release into BALF (Fig. 3f). These alterations were reversed by down-regulation of PTPRO. Analysis of BALF cell counts showed that PTPRO deficiency reduced HS-induced inflammatory cell infiltration, as evidenced by reduction in the number of total leukocyte, neutrophils and macrophage (Fig. 3g). Taken together, our studies indicated that HS-induced inflammation was mitigated when PTPRO was inhibited.

### Knockdown of PTPRO contributes to the suppression of the NF-κB signaling pathway activation in HS

**Fig. 4:**
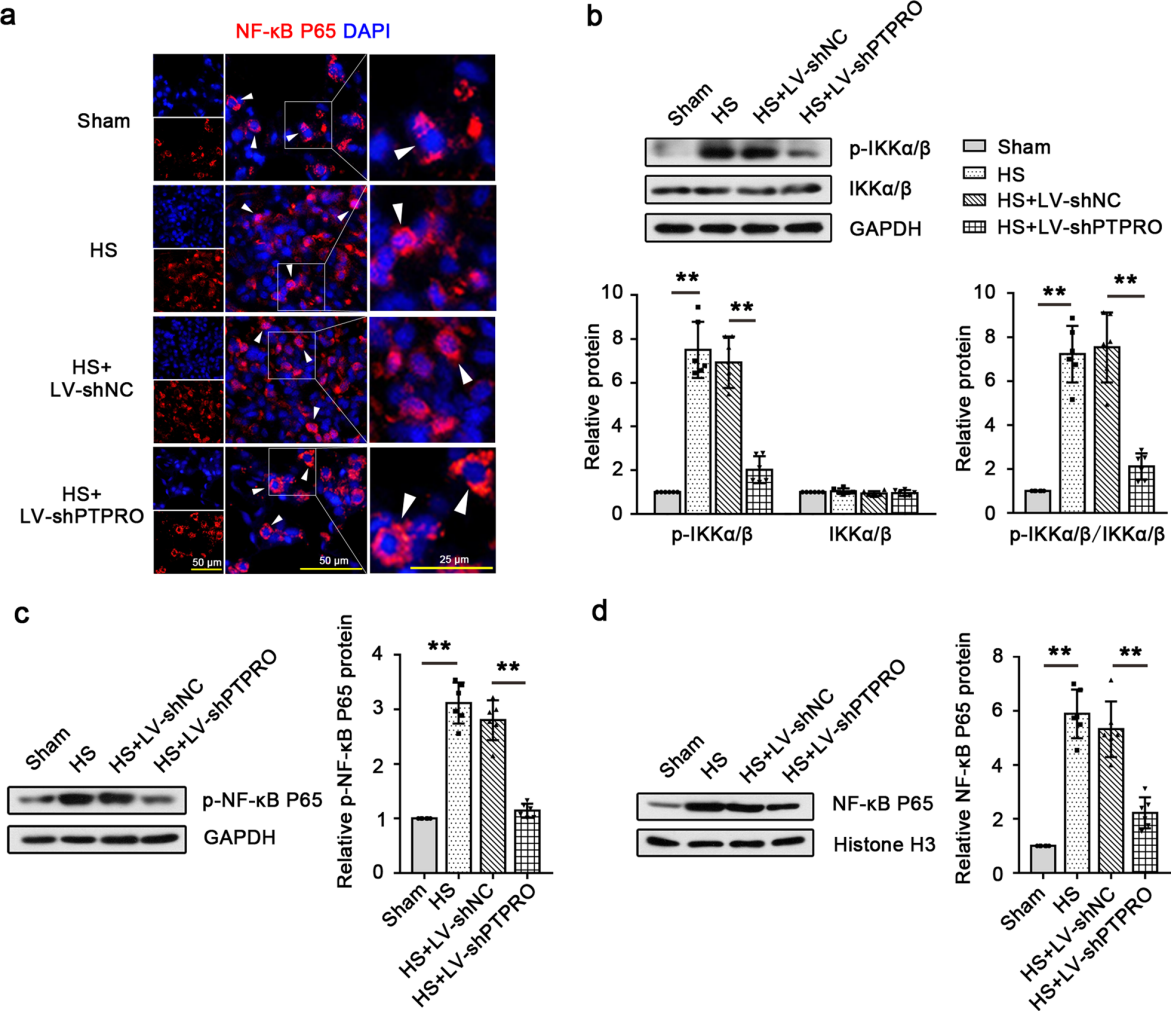
Knockdown of PTPRO contributes to the suppression of the NF-κB pathway activation in HS. **a** Immunofluorescence staining showed the intracellular localization of NF-κB P65 in the lung tissues. Magnification: 400 × . Scale = 50 μm or 25 μm. White arrow heads show the representative intracellular localization of NF-κB P65. **b** Expression levels of total IKKα/β and phosphorylated IKKα/β in the rat lung were detected by Western blot and the ratio of p-IKKα/β to total IKKα/β was calculated. **c, d** Western blot and expression quantification of phosphorylated NF-κB P65. **c** and nuclear NF-κB P65. **d** In the lungs were performed. Values are means (n = 6) ± SD. **P < 0.01

The expression of the proteins involved in the NF-κB signaling pathway was analyzed. Immunofluorescence staining revealed that NF-κB P65 was mainly expressed in the cytoplasm and HS promoted the translocation of NF-κB P65 from the cytoplasm into the nucleus. Down-regulation of PTPRO abolished the effect of HS on NF-κB P65 translocation (Fig. 4a). Western blot analysis showed that HS enhanced the p-IKKα/β level without altering total IKKα/β expression. The ratio of p-IKKα/β protein level to total IKKα/β level increased by HS dramatically declined when PTPRO was knocked down, which demonstrated that HS promoted IKKα/β phosphorylation while PTPRO knockdown restricted this process in the lungs (Fig. 4b). The level of phosphorylated NF-κB P65 increased by HS was also regressed by PTPRO down-regulation (Fig. 4c). Moreover, knockdown of PTPRO caused a decreased level of nuclear NF-κB P65 expression in the lungs of HS rats (Fig. 4d). PTPRO knockdown reduced the phosphorylation of IKKα/β and NF-κB P65 and inhibited the translocation of NF-κB P65 from the cytoplasm into the nucleus, thus restricting the activation of the NF-κB signaling pathway.

## Discussion

In this study, our results demonstrate that knockdown of PTPRO mitigates lung injury after HS by reducing inflammation, which may be associated with the suppression of the NF-κB signaling pathway.

The expression and function of PTPRO in the lungs after HS has not been explored yet. In the current study, we found that PTPRO was significantly up-regulated in the lung of HS rats. The expression of PTPRO truncated isoform (PTPROt) is increased in liver macrophages with the increasing degree of nonalcoholic steatohepatitis [25]. PTPRO is down-regulated in injured podocytes and lung squamous cell carcinoma compared with healthy tissues [26, 27]. Having observed abnormalities in PTPRO expression in multiple experimental models of human diseases, we hypothesize that PTPRO may play an important role in the pathogenesis of HS. This study confirmed that PTPRO knockdown alleviated HS-induced lung injury and improved lung barrier function. The therapeutic potential of PTPRO knockdown in lung injury indicates that PTPRO is likely to be a crucial driver to the progression of HS-induced lung injury.

Inflammation is a major event in the development of ALI following HS. KC and MIP-2 are neutrophil infiltration chemokine markers and MCP-1 is a specific chemokine marker of macrophage infiltration. Consistent with previous findings [28], the expression levels of inflammatory cytokines/chemokines (TNF-α, IL-6, MCP-1, KC, and MIP-2) were increased in the lungs after HS. These inflammatory cytokine/chemokine levels were reduced by inhibition of PTPRO. As a marker for neutrophil activation, MPO expression and activity in the injured lungs were reduced by PTPRO down-regulation. There were fewer inflammatory cells including total leukocyte, neutrophils, and macrophage in BALF of HS rats with PTPRO knockdown. These results revealed that PTPRO knockdown could inhibit inflammatory cell infiltration and the release of inflammatory cytokines/chemokines. A previous study suggests that PTPRO may contribute to inflammatory response in preeclampsia [16]. Thus, PTPRO may play an anti-inflammatory role in HS-induced ALI.

Inflammation is strongly associated with the NF-κB signaling pathway. It has been well established that suppression of the NF-κB pathway plays an anti-inflammatory role in lung injury [29, 30]. Recently, PTPRO was reported to exaggerate inflammation in vivo and in vitro through enhancing NF-κB activation [15, 31]. Based on these findings, we wondered if PTPRO regulates inflammation in HS via the NF-κB signaling pathway. As predicted, IKKα/β phosphorylation was reduced by PTPRO knockdown in the lungs after HS. It is IKKβ, not IKKα that is required for activation of the canonical NF-κB signaling pathway through activation of IκBs [32]. PTPRO down-regulation also restrained the NF-κB P65 phosphorylation and translocation of NF-κB P65 from the cytoplasm into the nucleus. These results indicate that PTPRO knockdown may suppress inflammation in HS-induced lung injury via inhibiting the NF-κB signaling pathway.

**Fig. 5:**
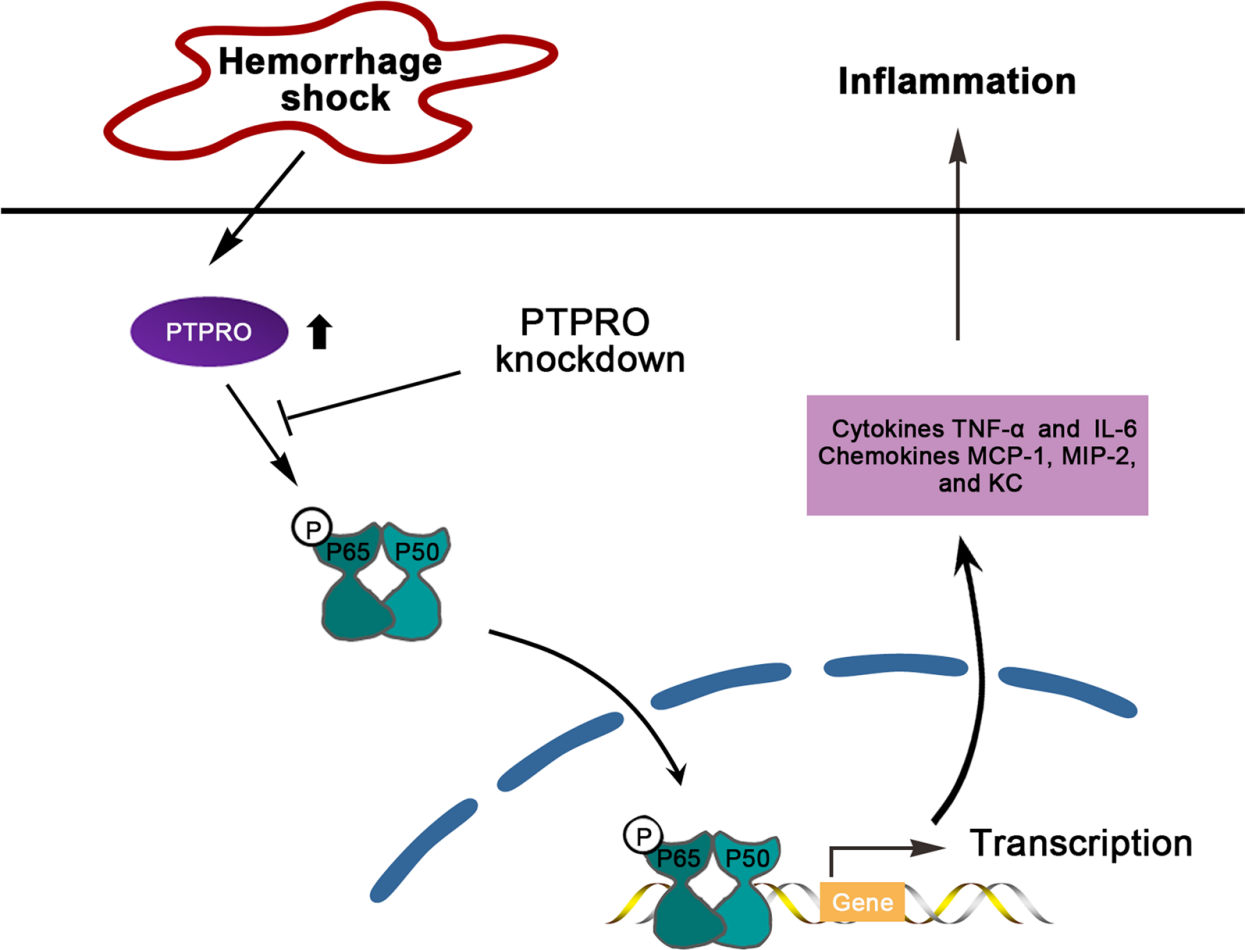
Diagram of the potential regulatory mechanism of PTPRO in HS-induced lung inflammation. PTPRO is up-regulated in the lungs after HS, which may contribute to NF-κB P65 phosphorylation and the translocation of NF-κB from the cytoplasm into the nucleus. Activated NF-κB in the nucleus likely initiates the transcription of proinflammatory genes including cytokine and chemokine, thus inducing lung inflammation following HS. PTPRO knockdown alleviates the HS-induced inflammation in the lungs by reducing the NF-κB pathway activation

NF-κB lies downstream of antigen receptors, growth factors, and cytokine receptors including TNF receptor family. A diverse range of stimuli including LPS, cytokines TNF-α, reactive oxygen species, and other factors can activate this dimer [33]. NF-κB P65 comprises the predominant NF-κB transcriptional activity and it is responsible for the expression of a large number of proinflammatory mediators [34]. In the current research (Fig. 5), HS induced the expression of PTPRO in the lungs, which may activate the NF-κB phosphorylation and its translocation from the cytoplasm into the nucleus, thus initiating the transcription of pro-inflammatory genes including cytokine TNF-α and IL-6, and chemokine MIP-2, MCP-1, and KC in the lungs. The recruitment and infiltration of macrophages and neutrophils triggered inflammation in the lungs. Inflammation and disruption of tight junctions between alveolar epithelial cells eventually lead to the breakdown of the epithelial barrier and enhanced lung permeability [35]. However, PTPRO knockdown attenuates HS-induced inflammation and lung injury as well as reduces the NF-κB pathway activation in HS, which suggests that PTPRO knockdown is likely to protect against HS-induced inflammation through inhibiting the NF-κB signaling pathway in the lungs. Nevertheless, in the present study, there was no more experiment able to confirm that PTPRO was involved in HS-induced lung inflammation via regulating the NF-κB signaling pathway. To make this finding more convincing, further exploration should be carried out in the future.

Damage control strategies, including early control of bleeding and balanced fluid resuscitation, are proposed as the standard of care for HS patients [36, 37]. Current trauma guidelines in humans recommend permissive hypotension (mean arterial pressure 50–60 mmHg) [38], recognizing the potentially harmful effects of rigorous fluid resuscitation. Several randomized controlled trials reported the benefits of limited fluid resuscitation or hypotensive resuscitation in the management of trauma patients with HS [39–41]. Scheiber et al. [42] showed higher survival of HS patients receiving restrictive versus standard volume replacement. In our model of HS, the hemorrhagic rats were resuscitated by an infusion of Lactated Ringer’s solution until the blood pressure is normalized. This animal model is widely used for studying HS in vivo in current research [21–23]. The procedure of fluid resuscitation needs to be carried out carefully and the fluid volume is critical, since aggressive fluid resuscitation could lead to fluid overload and, subsequently, to pulmonary edema (a component of acute lung injury). Of note, rats in each group received same volume of fluids during resuscitation. Thus, the influence of resuscitation and the lung injury induced by HS were consistent between groups in this study. The lung injury following HS and resuscitation may be not only due to hemorrhage but also fluid resuscitation. The potentially harmful effects of fluid resuscitation on HS deserve further exploration in the future.

In conclusion, we demonstrate that PTPRO knockdown plays an anti-inflammatory role in HS-induced lung injury, which is associated with its inhibitory effect on NF-κB pathway activation. This finding indicates that PTPRO may be a potential therapeutic target for ameliorating lung injury in patients suffering from HS.

## Supplementary Information

**Additional file 1**: **Table S1**. Patient Characteristics.

## Data Availability

The data sets used and/or analyzed during this study are available from the corresponding author upon reasonable request.
