# Supplementary material for: PTPRO knockdown protects against inflammation in hemorrhage shock-induced lung injury involving the NF-κB signaling pathway
Source: Respir Res. 2022 Jul 29;23:195. doi: 10.1186/s12931-022-02118-2 (PMC9335982; doi:10.1186/s12931-022-02118-2)
Supplement: Supplementary file 1 — Additional file 1: Table S1. Patient Characteristics. [file 12931_2022_2118_MOESM1_ESM.docx]

**Supplementary Table 1**. Patient Characteristics.

| Trauma-Associated Severe Hemorrhage Score (Points) | 15 | 14 | 15 | 12 | 14 | 12 | 14 |
| --- | --- | --- | --- | --- | --- | --- | --- |
| Injury Severity Score (points) | 26 | 13 | 17 | 17 | 17 | 30 | 26 |
| Hemoglobin level(g/L) | 79 | 57 | 73 | 78 | 66 | 71 | 57 |
| Respiratory rate (breaths/min) | 22 | 20 | 23 | 22 | 20 | 20 | 21 |
| Heart rate (beats/min) | 117 | 93 | 92 | 115 | 97 | 110 | 97 |
| Diastolic pressure (mm Hg) | 59 | 58 | 54 | 44 | 60 | 57 | 52 |
| Systolic pressure (mm Hg) | 89 | 72 | 88 | 85 | 89 | 89 | 84 |
| Glasgow Coma Scale score, median (IQR)<8 | 14 | 15 | 14 | 14 | 15 | 15 | 15 |
| 24-h Mortality | None | None | None | None | None | None | None |
| Complication | None | None | None | None | None | None | None |
| Type of injury | Left femoral fracture | Right lower limb open facture | Left femoral open fracture | Right upper arm severed | Forearms severed | Lower limb polytrauma | Right arm severed |
| Age (years) | 33 | 19 | 64 | 70 | 33 | 37 | 57 |
| Sex | Male | Male | Male | Male | Male | Male | Male |
| Patient | 1 | 2 | 3 | 4 | 5 | 6 | 7 |
